# *Polygonatum sibiricum* Polysaccharides Extracted with Ultrasound-Assisted Deep Eutectic Solvents Protect L6 Cells Against Oxidative Stress in a Cellular Model of Sarcopenic Obesity

**DOI:** 10.3390/antiox14030315

**Published:** 2025-03-06

**Authors:** Chaoqun Sun, Yifan Xu, Hanchen Du, Yan Chen, Wenjie Qu, Menglu He, Zhengyi Liu, Jian Huang, Junsheng Huo, Jiyong Yin, Jing Liu

**Affiliations:** 1 Key Laboratory of Public Nutrition and Health, National Health Commission of the People’s Republic of China, National Institute for Nutrition and Health, Chinese Center for Disease Control and Prevention, Beijing 100050, China; sunchaoqun97@163.com (C.S.); xuyf@ninh.chinacdc.cn (Y.X.); duhc@ninh.chinacdc.cn (H.D.); chenyan7341@163.com (Y.C.); qwj370612@163.com (W.Q.); heml@ninh.chinacdc.cn (M.H.); liuzy@ninh.chinacdc.cn (Z.L.); huangjian@ninh.chinacdc.cn (J.H.); huojs@ninh.chinacdc.cn (J.H.); 2 Space Biological Proactive Health Research Institute, Beijing 100043, China

**Keywords:** *Polygoatum sibiricum* polysaccharide, oxidative stress, L6 cell, ultrasound-assisted deep eutectic solvents, sarcopenia obesity

## Abstract

Oxidative stress is closely associated with sarcopenia obesity (SO). As the primary active component of *Polygonatum sibiricum*, *Polygonatum sibiricum* polysaccharide (PsP) is recognized as a potent antioxidant and has become a focus of research for potential therapeutic strategies against SO. Our previous study demonstrated that ultrasound-assisted deep eutectic solvents (UAE-DESs) significantly improve the extraction efficiency of PsP; however, the antioxidant effect of PsP extracted using UAE-DESs was unexplored. This study investigated the effects of PsP extracted by UAE-DESs on an oxidative stress model in L6 cells induced by palmitic acid (PA). The results revealed that PsP enhanced the ability of L6 cells to resist PA-induced effects, including ectopic lipid deposition, changes in reactive oxygen species (ROS) levels, malondialdehyde (MDA) contents, and the activities of glutathione peroxidase (GSH-Px), superoxide dismutase (SOD), and catalase (CAT). Additionally, PsP upregulated the expression of myosin heavy chain (MHC) and myoblast differentiation (MyoD) protein, while increasing myotube cell diameter. These findings suggested that PsP extracted by UAE-DESs can enhance the antioxidant capacity of L6 cells against PA-induced oxidative stress in a simulated SO model, providing a potential therapeutic agent for the prevention and treatment of SO.

## 1. Introduction

*Polygoatum sibiricum* Delar. ex Redoute, an ingredient in traditional Chinese food and medicine, comprises over 60 varieties worldwide, with more than 30 varieties found in China alone [1,2]. These varieties are widely recognized for their exceptional nutritional and medicinal properties, making them a valuable focus of research in both food science and traditional medicine. Modern pharmacological studies have demonstrated that *Polygoatum sibiricum* exhibits a wide range of pharmacological activities, including anti-oxidative effects [3], anti-aging effects [4], the potential for preventing and treating senile dementia [5,6], hypoglycemic effects [7,8], anti-tumor properties [9,10] and so on. Zhao et al. [11] have showed that the biological efficacy of *Polygoatum sibiricum* is primarily attributed to its rich functional components, such as polysaccharides, saponins, flavonoids, polyphenols and so on.

*Polygoatum sibiricum* polysaccharide (PsP) is not only the primary carbohydrate component of *Polygoatum sibiricum* but also its main active ingredient [12]. It holds a significant position in traditional medicine and functional food development. Maximizing the utilization of PsP resources is of great value and significance. Therefore, research on the biological activity of PsP that employs novel extraction techniques is crucial for advancing its application and development in functional food.

As the primary active component of *Polygoatum sibiricum*, PsP has been shown to enhance immunity, treat diabetes, inhibit tumor growth [13,14], and exhibit antioxidant properties by scavenging free radicals and regulating the activity of enzymes involved in oxidative defense [15]. Tural et al. [16,17] demonstrated that PsP intervention significantly reduced malondialdehyde (MDA) levels in serum and skeletal muscle while increasing the activity of three key antioxidant enzymes. Wang et al. [18] further confirmed the specific antioxidant capacity of PsP at certain concentrations through in vitro antioxidant assays. These findings substantiate the antioxidant activity of PsP and provide a critical scientific foundation for its application in health products and related fields.

Oxidative stress is a cellular imbalance that can damage biological macromolecules, such as proteins, lipids, and nucleic acids, ultimately leading to cellular dysfunction and even apoptosis [19,20]. Ectopic lipid deposition-induced oxidative stress is one of the key contributors to sarcopenic obesity (SO) [21]. Therefore, research on enhancing cellular antioxidant capacity, particularly strategies to strengthen protective mechanisms before oxidative stress occurs, is critical for mitigating cellular damage and preserving cellular function. Such studies provide a foundation for addressing SO. Beetham et al. [22,23] demonstrated that excessive palmitic acid (PA) intake can induce intracellular ectopic lipid deposition, subsequently triggering oxidative stress. PA is commonly used to establish oxidative stress models in cells. Rat L6 cells, a widely utilized muscle cell line, play a significant role in studying lipid metabolism and oxidative stress, making them a preferred choice for constructing oxidative stress models [24]. When L6 cells are exposed to excessive PA, they absorb and accumulate large amounts of lipids, leading to significant lipid deposition. As lipid deposition increases, higher levels of oxidative stress markers, such as reactive oxygen species (ROS), are generated within the cells. These ectopically deposited lipids and oxidative stress markers disrupt normal cellular metabolism, impair cellular function and structure, and ultimately contribute to cellular deterioration and apoptosis [25]. Thus, the PA-induced oxidative stress model in L6 cells serves as a valuable cellular model for simulating SO [26].

Extraction by ultrasound-assisted deep eutectic solvents (UAE-DESs) is an emerging extraction technique that integrates the principles of deep eutectic solvent extraction with ultrasound-assisted extraction [27,28]. This innovative approach leverages the synergistic advantages of both methods, combining the enhanced mass transfer and cell disruption capabilities of ultrasound with the tunable solvent properties of deep eutectic systems. Notably, UAE-DESs not only preserves the bioactivity of target compounds but also significantly enhances extraction efficiency compared to conventional methods [29]. These characteristics make UAE-DESs a promising and effective technique for the extraction of bioactive components from various biological matrices.

Our previous study demonstrated that the UAE-DESs method significantly enhances the extraction efficiency of PsP, achieving an extraction yield of 43.61% [30]. However, the biological activity of PsPs extracted using the UAE-DESs method still needs to be thoroughly validated. Therefore, comprehensive biological investigations are required to confirm whether the UAE-DESs method can maintain the bioactivity of PsP while improving its extraction efficiency.

In the present study, we employed a palmitic acid (PA)-induced oxidative stress model in L6 cells to evaluate the potential antioxidant effects of PsP extracted using the UAE-DESs method. This investigation aims to demonstrate that UAE-DESs not only enhances PsP extraction efficiency but also preserves its antioxidant properties. Furthermore, through this experimental approach, we seek to establish that the UAE-DESs extraction process does not compromise the functional integrity of PsP, particularly its antioxidant capacity as a polysaccharide. Additionally, this study may provide novel insights into the potential application of PsP in the prevention and treatment of sarcopenic obesity (SO), as current research lacks substantial evidence regarding the therapeutic effects of PsP on SO-related conditions.

## 2. Materials and Methods

### 2.1. Materials

The roots of *Polygoatum sibiricum* were collected from Shunyi District (Beijing, China). The rat L6 skeletal muscle cell line was obtained from the Cell bank of the Chinese Academy of Sciences Co., Ltd. (Wuhan, China). All chemical reagents and materials used in this study are listed in Table 1.

### 2.2. Main Instruments and Equipment

The following equipment was utilized in the experiment: A1 Laser Scanning Confocal Microscope (Nikon Corporation Co., Ltd., Tokyo, Japan); EVOS-M7000 Fluorescence Microscope (Thermo Fisher Scientific, Inc. Waltham, MA, USA); MCO-18AIC CO2 Incubator (Panasonic Corporation Co., Ltd., Tokyo, Japan); Allegra x-22 R Centrifuge (Beckman Coulter, Inc., Brea, CA, USA); SpectraMax I3X Enzyme marker (Molecular Devices Instruments Ltd., San Jose, CA, USA); Desk centrifuge 5418 (Eppendorf, Inc., Hamburg, Germany); Vortex mixing device ORTEx Genius (IKA, Inc., Staufen, Germany); Digital ultrasonic cleaner (Kunshan Ultrasonic Instruments Co., Ltd., Kunshan, China); Magnetic Stirrers (IKA, Inc., Staufen, Germany); U-3900 Spectrophotometer (Hitachi, Ltd., Tokyo, Japan); Circulating water multi-purpose vacuum pump (Gongyi Yuhua Instrument Co., Ltd., Henan, China); Freeze dryer (Ningbo Scientz Biotechnology Co., Ltd., Zhejiang, China); and Rotary evaporator (Shanghai Xiande Experimental Instrument Co., Ltd., Shanghai, China).

### 2.3. Preparations of the PsP Solutions of Different Dosages and Positive Control Solution

The *Polygonatum sibiricum* roots were dried to a constant weight in an oven at 45 °C and subsequently ground to pass through an 80-micron mesh sieve in order to obtain a fine powder. A precisely measured quantity of the powder was transferred into a centrifuge tube, and a deep eutectic solvent was added at a predetermined liquid-to-material ratio. The mixture was then subjected to ultrasound-assisted extraction under optimized time and temperature conditions. Following extraction, the PsP was precipitated by adding 80% ethanol and incubating the solution at 4 °C for 12 h. The precipitate was collected by centrifugation, and the extraction yield of PsP was quantified using the phenol–sulfuric acid method. The PsP concentration (C, mg/mL) was determined in triplicate to ensure accuracy. To purify the extracts, proteins were removed using the Sevage method, and the solution was dialyzed using a 1000 Da molecular weight-cutoff dialysis bag. The dialysis process was performed in distilled water on a magnetic stirrer, with the water replaced every 24 h over a period of 48 h. After dialysis, residual impurities and small molecules were further eliminated by centrifugation. The purity of the PsP was confirmed to be 98% prior to use. For experimental applications, 50 mg of PsP extracted via the UAE-DESs method [30] was dissolved in 5 mL of distilled water to prepare a 5 mg/mL solution. The solution was sterilized by heating it in a water bath at 70 °C for 20 min and subsequently stored at 4 °C for further use.

A total of 1 g N-acetyl-L-cysteine (NAC) was dissolved in 12.256 mL of deionized water, and it was mixed well after complete dissolution. Then, a 0.22 μm filter membrane was used to filter it, so as to obtain the sterile 500 mM NAC stock solution, which was stored at −20 °C.

### 2.4. Cell Culture and Treatment

This study employed an experimental design in which PsP intervention was administered prior to establishing a PA-induced L6 cell model simulating SO. PsP intervention was initiated 6 h before PA induction. The experimental setup included a model group, a negative control group, a positive control group, and five dose groups, each with triplicate wells to ensure reproducibility. L6 cells were seeded into a 96-well plate at a density of 1 × 10^5^ cells per well under optimal growth conditions. Subsequently, cell differentiation was induced using a differentiation medium consisting of DMEM supplemented with 2% horse serum and 1% penicillin-streptomycin (double antibiotic).

Following myotube formation, the differentiation medium was administered as follows: the model group and negative control group received the standard differentiation medium, while the positive control group was treated with a differentiation medium supplemented with 5 mM NAC. The five dose groups were treated with differentiation media containing varying concentrations of PsP extracted using the UAE-DESs method. After the intervention, all cells were washed twice with sterile phosphate-buffered saline (PBS). Subsequently, the model group, positive control group, and PsP dose groups were exposed to differentiation medium containing 0.75 mM PA, whereas the negative control group received standard differentiation medium. All groups were then cultured for 48 h to complete the model establishment. Upon completion of the modeling process, cells from each group were harvested for subsequent analysis. Depending on the requirements of the follow-up experiments, cells were either subjected to specific indicator measurements or prepared for microscopic observation. The detailed intervention and modeling protocols are summarized in Table 2.

### 2.5. Cell Viability Assay

Following the intervention experiment, L6 cells from each group were washed twice with sterile PBS and subsequently cultured in serum-free DMEM medium for 1 h. After the incubation period, 10 μL of CCK-8 solution was added to each well, and the cells were incubated at 37 °C for 30 min. Absorbance was measured at 450 nm using an enzyme marker. The results were expressed as a percentage relative to the negative control group. Cell viability activity was calculated using the following formula:

$$P(\%)=(\frac{Aj-Ai}{Ao-Ai})\times 100$$

*P*: cell proliferation rate; Aj: experimental group; Ai: blank group; and Ao: negative control group.

### 2.6. Triglyceride Assay

After normalizing the protein concentrations of the samples using the bicinchoninic acid assay (BCA) method, 2.5 μL of each sample and triglyceride (TG) standard were added to a 96-well plate. Following the kit instructions, 250 μL of working solution was added to each well, with distilled water serving as the blank. The plate was incubated at 37 °C for 10 min, after which the absorbance of each well was measured at 510 nm using an enzyme marker. The TG levels were calculated based on the protein concentrations of the cells and expressed in mmol/g.

### 2.7. Oil Red O Staining

After the washing of the L6 cells of each group twice with sterile PBS, 4% paraformaldehyde was added to each well and allowed to fix the cells for 30 min. Following fixation, each well was rinsed three times with distilled water. The cells were then treated with 60% isopropanol for 30 s and stained with 5% oil red O solution for 30 min. Subsequently, the wells were rinsed with 60% isopropanol for 30 s, and washed with distilled water until the background became transparent. Finally, nuclei were counterstained with hematoxylin for 4 min, and images were captured using a microscope. The results were analyzed and compared across groups, according to the experimental design.

### 2.8. ROS Assay

After the culturing and grouping of the L6 cells, they were incubated at 37 °C in their original culture medium, supplemented with 10 μM 2′,7′-dichlorodihydrofluorescein diacetate (DCFH-DA), for 20 min. Following incubation, the cells were washed three times with serum-free DMEM medium. The fluorescence intensity of each group was measured using excitation and emission wavelengths of 488 nm and 525 nm, respectively, and the signal values were recorded. The results were expressed as a percentage relative to the negative control group.

### 2.9. Glutathione Peroxidase Activity Assay

The glutathione peroxidase (GSH-Px) activity was measured using a commercial assay kit, following the manufacturer’s instructions for the preparation of the detection solution and reaction initiation solution. The experiment was conducted using blank wells, control wells, and sample wells, according to the experimental design. After the sequential addition of the detection buffer, sample solution, and detection working solution to each well, 4 μL of reaction initiation solution was added. Absorbance was measured at 450 nm under the following conditions: a total duration of 20 min, with measurements taken at 4 min intervals, and a constant temperature of 25 °C. GSH-Px activity was calculated using the following formula:

$$A(mU/mg)=\frac{Aj-Ai}{0.00622\times P}$$

*A*: glutathione peroxidase activity; Aj: sample group; Ai: blank group; and *P*: protein concentration.

### 2.10. Superoxide Dismutase Activity Assay

The protein concentrations of the samples were normalized using the BCA method. Following the kit’s instructions, an appropriate amount of WST-8/ enzyme working solution was prepared, and the reaction initiation solution was diluted with detection buffer. The experiment was conducted using blank wells 1,2,3, control wells, and sample wells, according to the experimental design. Samples and other solutions were added sequentially, and the L6 cells were incubated at 37 °C for 30 min. Absorbance was measured at 450 nm, and the signal values of each group were recorded to calculate the superoxide dismutase (SOD) activity. The SOD activity was calculated using the following formula:

$$Y\left(\%\right)=\frac{\left(A1-A2\right)-\left(Aj-A3\right)}{\left(A1-A2\right)}\times 100$$

*Y*: inhibition percentage; Aj: sample group; A1: blank group 1; A2: blank group 2; and A3: blank group 3.

$$A(U/mg)=\frac{Y}{\left(1-Y\right)\times P}$$

*A*: superoxide dismutase activity; *Y*: inhibition percentage; and *P*: protein concentration.

### 2.11. Catalase Activity Assay

Prior to measurement, the working solution from the catalase (CAT) detection kit was pre-warmed in a water bath at 37 °C for 10 min. A 1 mL aliquot of the working solution was transferred to a 1 mL quartz cuvette; this was followed by the addition of 35 μL of the sample. The mixture was gently mixed for 5 s. The initial absorbance (A1) at 240 nm was measured immediately, and the absorbance (A2) was recorded after 1 min at room temperature. The difference in absorbance (ΔA) was calculated as ΔA = A1 − A2. CAT activity was determined using the following formula:

$$A(U/mg)=\frac{678\times \Delta A}{P}$$

*A*: catalase activity; *P*: protein concentration.

### 2.12. MDA Assay

An appropriate amount of testing solution was prepared according to the kit instructions, and the standard was serially diluted to various concentrations using distilled water. Subsequently, 0.1 mL of the sample solution and standard solution were added to separate centrifuge tubes; this was followed by the addition of 0.2 mL of detection working solution to each tube. The tubes were then incubated in a water bath at 100 °C for 15 min. After cooling to room temperature, the tubes were centrifuged at 2500 rpm for 15 min, and the supernatant was collected. Absorbance was measured at 532 nm, and the signal values for each group were recorded. The MDA content of each sample was calculated based on the standard curve.

### 2.13. Diameter of Myotube Cells Assay

Following the modeling process, images of each group were captured using a confocal microscope at a magnification of 40×. For each well, four random visual fields were selected, and within each field, four myotubes were randomly chosen for diameter measurement. The diameter of the L6 myotubes was measured, using NIS-Elements Advanced Research 5.41.02 software, by a researcher blinded to the experimental groups. The myotube diameters for each group were recorded, and the results were expressed as a percentage relative to the negative control group.

### 2.14. Myosin Heavy Chain Protein Expression-Level Assay

The expression levels of myosin heavy chain (MHC) protein in each group were determined using an MHC ELISA kit, following the manufacturer’s instructions. Diluted samples and standards are added to the wells and incubated, allowing the MHC proteins to bind to the capture antibody. A biotinylated detection antibody specific to a different epitope on the MHC protein was added; this was followed by incubation and washing. Streptavidin conjugated to horseradish peroxidase (HRP) was added, binding to the biotinylated detection antibody. A chromogenic substrate was then added which reacts with HRP to produce a color change. The reaction was finally halted with a stop solution, the absorbance was measured at 450 nm, and the signal values for each group were recorded. The MHC protein expression levels were calculated based on the standard curve.

### 2.15. Myoblast Differentiation Protein Expression-Level Assay

The expression levels of the myogenic differentiation (MyoD) protein in each group were determined using a MyoD ELISA kit, following the manufacturer’s instructions. Diluted samples and standards were added to the wells and incubated, allowing MyoD proteins to bind to the capture antibody. A biotinylated detection antibody specific to a different epitope on the MyoD protein was added; this was followed by incubation and washing. Streptavidin conjugated to horseradish peroxidase (HRP) was added, binding to the biotinylated detection antibody. A chromogenic substrate was then added which reacts with HRP to produce a color change. The reaction was finally halted with a stop solution, the absorbance was measured at 450 nm, and the signal values for each group were recorded. The MyoD protein expression levels were calculated based on the standard curve.

### 2.16. Statistical Analysis

All experiments were conducted in strict accordance with the research design, with each experimental group repeated in triplicate. The data were assessed for normal distribution using the Shapiro–Wilk test and for homogeneity of variance using an appropriate statistical test. The results are presented as mean ± standard deviation (M ± SD). Statistical analysis was performed using one-way analysis of variance (ANOVA), followed by the least significant difference (LSD) post-hoc test to evaluate the effects of different intervention schemes. A *p*-value < 0.05 was considered statistically significant. All statistical analyses were conducted using SPSS19.0 software (IBM, Armonk, NY, USA), and graphs were generated using Microsoft Excel 2010 software (Microsoft, Redmond, WA, USA).

## 3. Results

The results demonstrated significant differences, in all measured indicators, between the model group and the negative control group, confirming the successful establishment of the PA-induced oxidative stress model in the L6 myotube cells.

### 3.1. Cell Proliferation Activity Assay

**Figure 1:**
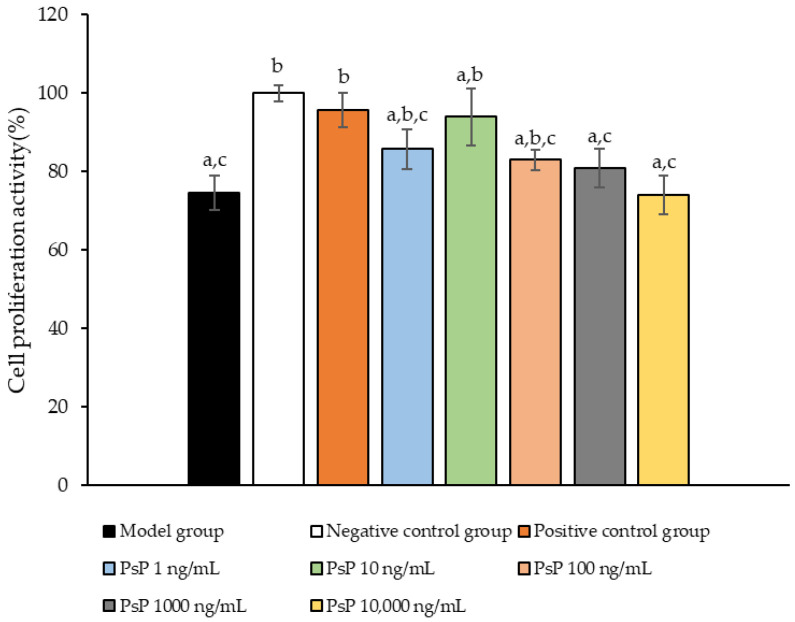
Measurement results for L6 cell proliferation activity. Note: The superscript “a” indicates a statistically significant difference compared to the negative control group (*p* < 0.05); “b” denotes a statistically significant difference compared to the model group (*p* < 0.05); and “c” represents a statistically significant difference compared to the positive control group (*p* < 0.05).

The comparison of cell proliferation activity levels among the experimental groups is presented in Figure 1. The cell activity levels in the three dose groups (1 ng/mL, 10 ng/mL, and 100 ng/mL) were significantly higher than model group (*p* < 0.05), indicating that PsP at concentrations of 1 ng/mL, 10 ng/mL, and 100 ng/mL enhanced the resistance of L6 cells to PA-induced oxidative stress. Among these, the 10 ng/mL dose demonstrated the most pronounced effect. These results suggest that PsP extracted using the UAE-DESs method, at appropriate concentrations, can effectively improve the proliferation activity of L6 myotube cells under PA-induced oxidative stress conditions.

### 3.2. TG Content Determination

**Figure 2:**
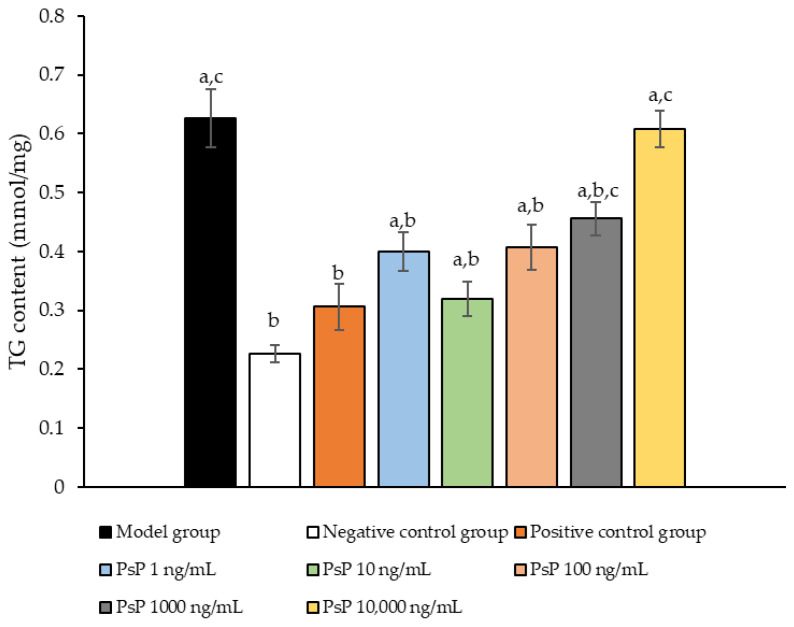
TG measurement results for L6 cells. Note: The superscript “a” indicates a statistically significant difference compared to the negative control group (*p* < 0.05); “b” denotes a statistically significant difference compared to the model group (*p* < 0.05); and “c” represents a statistically significant difference compared to the positive control group (*p* < 0.05).

The comparison of TG content levels among the experimental groups is presented in Figure 2. The TG content levels in the four intervention groups (1 ng/mL, 10 ng/mL, 100 ng/mL, and 1000 ng/mL) were significantly lower than model group (*p* < 0.05), demonstrating that PsP at concentrations of 1 ng/mL, 10 ng/mL, 100 ng/mL, and 1000 ng/mL effectively enhanced the resistance of L6 cells to PA-induced TG accumulation. Among these, the 10 ng/mL dose showed the most pronounced effect. These results suggested that PsP extracted using the UAE-DESs method retains its ability to mitigate PA-induced oxidative stress in L6 myotube cells, at specific concentrations.

### 3.3. Oil Red O Staining Result

**Figure 3:**
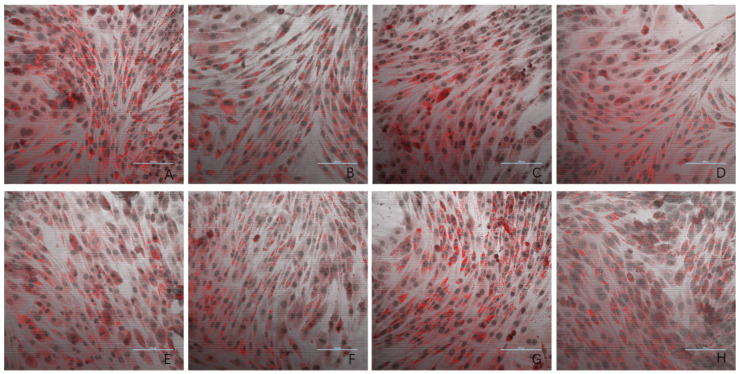
The effects of PA-induced lipid deposition in L6 myotube cells (oil red O × 40). Note: (**A**) model group; (**B**) negative control group; (**C**) 5 mM NAC group; (**D**) 1 ng/mL dose group; (**E**) 10 ng/mL dose group; (**F**) 100 ng/mL dose group; (**G**) 1000 ng/mL dose group; and (**H**) 10,000 ng/mL dose group.

The results of the oil red O staining for the eight experimental groups are presented in Figure 3. Compared to the negative control group, the model group exhibited a significantly higher number of lipid droplets in L6 cells. The three dose groups (1 ng/mL, 10 ng/mL, and 100 ng/mL) showed a marked reduction in lipid droplet accumulation compared to the model group. In contrast, the 1000 ng/mL and 10,000 ng/mL dose groups displayed lipid droplet levels similar to that observed in the model group. These findings provide evidence that PsP extracted using the UAE-DESs method can enhance the ability of L6 myotube cells to resist PA-induced ectopic lipid deposition, but only within a specific concentration range.

### 3.4. Determination of ROS Levels

**Figure 4:**
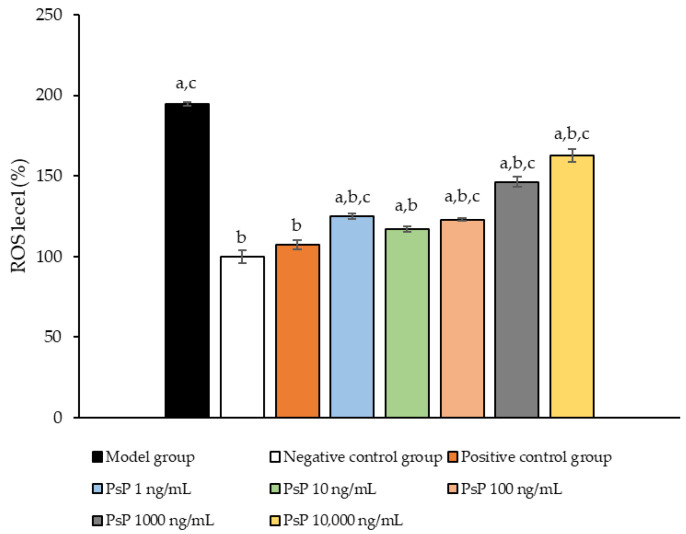
ROS measurement results in L6 cells. Note: The superscript “a” indicates a statistically significant difference compared to the negative control group (*p* < 0.05); “b” denotes a statistically significant difference compared to the model group (*p* < 0.05); and “c” represents a statistically significant difference compared to the positive control group (*p* < 0.05).

Figure 4 presents the comparison of ROS levels among the experimental groups. The ROS levels in the five groups (1 ng/mL, 10 ng/mL, 100 ng/mL, 1000 ng/mL, and 10,000 ng/mL) were significantly lower than model group (*p* < 0.05), indicating that PsP at these concentrations enhanced the resistance of L6 myotube cells to PA-induced ROS generation. Among these, the 10 ng/mL dose demonstrated the most pronounced effect. These results suggested that PsP extracted using the UAE-DESs method can effectively mitigate PA-induced oxidative stress in L6 myotube cells, within a specific concentration range.

### 3.5. Determination of GSH-Px Activity

**Figure 5:**
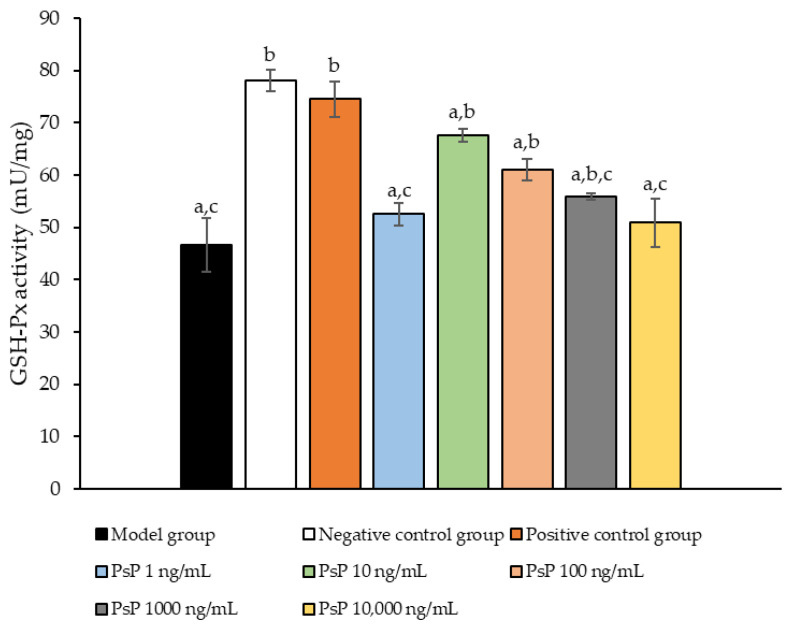
GSH-Px activity measurement results in L6 cells. Note: The superscript “a” indicates a statistically significant difference compared to the negative control group (*p* < 0.05); “b” denotes a statistically significant difference compared to the model group (*p* < 0.05); and “c” represents a statistically significant difference compared to the positive control group (*p* < 0.05).

Figure 5 presents the comparison of the GSH-Px activity levels among the experimental groups. The GSH-Px activity levles in the four dose groups (1 ng/mL, 10 ng/mL, 100 ng/mL, and 1000 ng/mL) were significantly higher than model group (*p* < 0.05), demonstrating that PsP at these concentrations enhanced GSH-Px activity in L6 myotube cells. Among these, the 10 ng/mL dose showed the most pronounced effect. These results suggest that PsP extracted using the UAE-DESs method can effectively alleviate PA-induced oxidative stress in L6 myotube cells, within a specific concentration range.

### 3.6. Determination of SOD Activity

**Figure 6:**
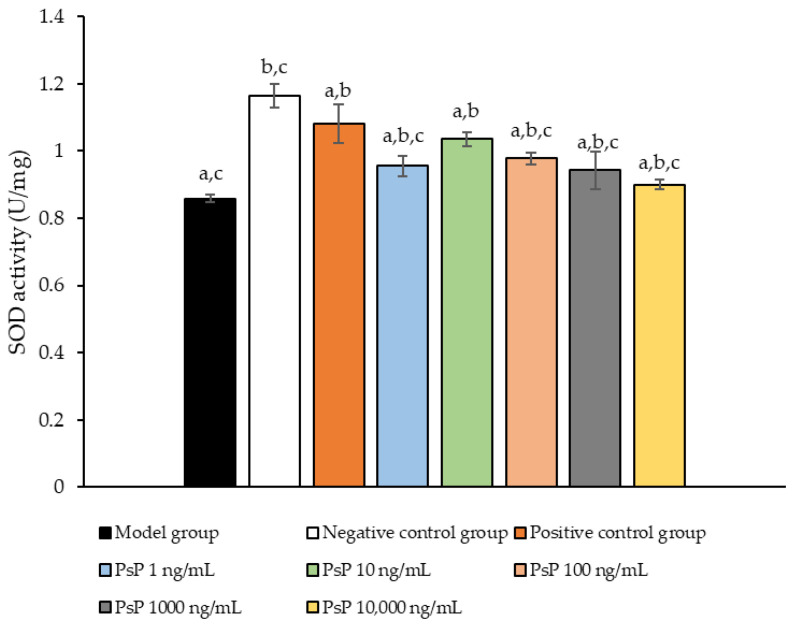
SOD activity measurement results in L6 cells. Note: The superscript “a” indicates a statistically significant difference compared to the negative control group (*p* < 0.05); “b” denotes a statistically significant difference compared to the model group (*p* < 0.05); and “c” represents a statistically significant difference compared to the positive control group (*p* < 0.05).

Figure 6 presents the comparison of SOD activity levels among the experimental groups. The SOD activities in the five dose groups (1 ng/mL, 10 ng/mL, 100 ng/mL, 1000 ng/mL, and 10,000 ng/mL) were significantly higher than model group (*p* < 0.05), indicating that PsP at these concentrations enhanced SOD activity in L6 myotube cells. Among these, the 10 ng/mL dose demonstrated the most pronounced effect. These results suggest that PsP extracted using the UAE-DESs method can effectively mitigate PA-induced oxidative stress in L6 myotube cells, within a specific concentration range.

### 3.7. Determination of CAT Activity

**Figure 7:**
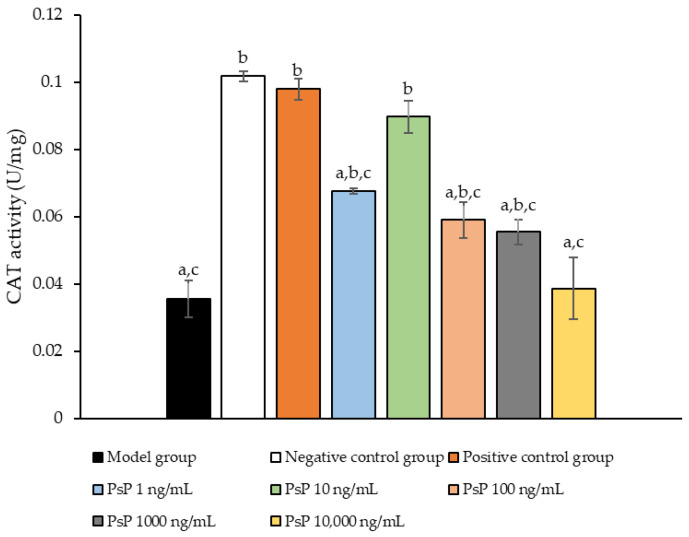
Catalase activity measurement results in L6 cells. Note: The superscript “a” indicates a statistically significant difference compared to the negative control group (*p* < 0.05); “b” denotes a statistically significant difference compared to the model group (*p* < 0.05); and “c” represents a statistically significant difference compared to the positive control group (*p* < 0.05).

Figure 7 presents the comparison of CAT activity among the experimental groups. The CAT activity levels in the four dose groups (1 ng/mL, 10 ng/mL, 100 ng/mL, and 1000 ng/mL) were significantly higher than model group (*p* < 0.05), demonstrating that PsP at these concentrations enhanced CAT activity in L6 myotube cells. Among these, the 10 ng/mL dose showed the most pronounced effect. These results suggest that PsP extracted using the UAE-DESs method can effectively alleviate PA-induced oxidative stress in L6 myotube cells, within a specific concentration range.

### 3.8. Determination of MDA Content

**Figure 8:**
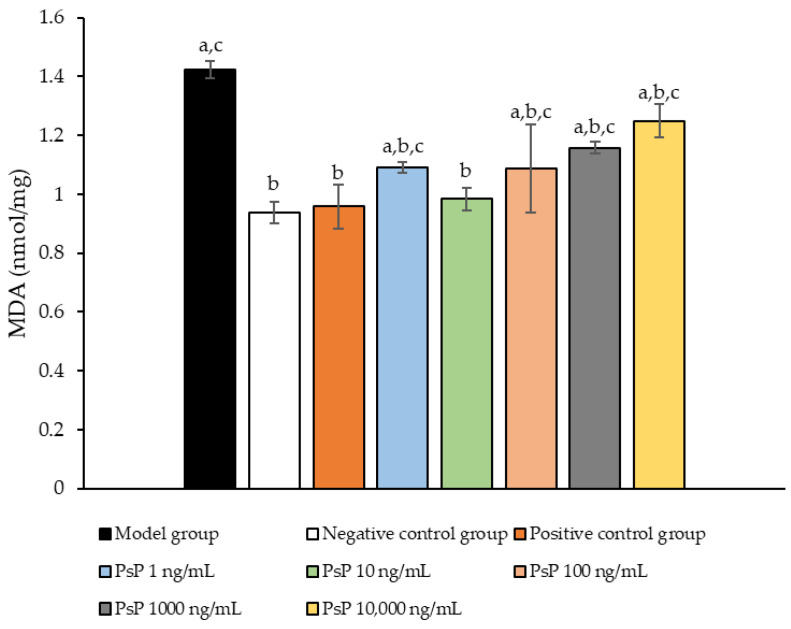
Determination of malondialdehyde in L6 cells. Note: The superscript “a” indicates a statistically significant difference compared to the negative control group (*p* < 0.05); “b” denotes a statistically significant difference compared to the model group (*p* < 0.05); and “c” represents a statistically significant difference compared to the positive control group (*p* < 0.05).

Figure 8 presents the comparison of MDA content levels among the experimental groups. The MDA levels in the five dose groups (1 ng/mL, 10 ng/mL, 100 ng/mL, 1000 ng/mL, and 10,000 ng/mL) were significantly lower than model group (*p* < 0.05), indicating that PsP at these concentrations enhanced the resistance of L6 myotube cells to PA-induced oxidative stress. Among these, the 10 ng/mL dose demonstrated the most pronounced effect. These results suggest that PsP extracted using the UAE-DESs method can effectively mitigate PA-induced oxidative stress in L6 myotube cells, within a specific concentration range.

### 3.9. Measurement of Myotube Cell Diameter

**Figure 9:**
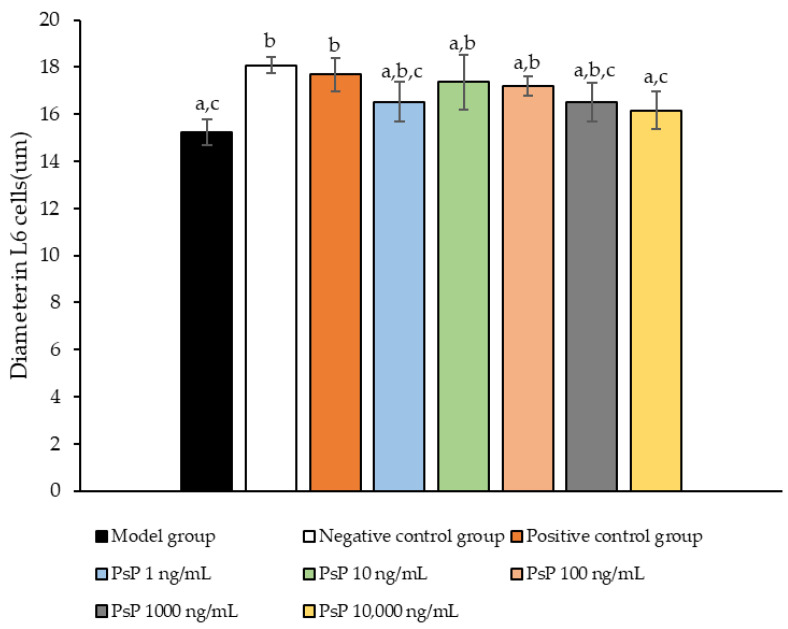
Determination of L6 myotube cell diameters. Note: The superscript “a” indicates a statistically significant difference compared to the negative control group (*p* < 0.05); “b” denotes a statistically significant difference compared to the model group (*p* < 0.05); and “c” represents a statistically significant difference compared to the positive control group (*p* < 0.05).

Figure 9 presents a comparison of myotube cell diameters across different experimental groups. The results demonstrate that the myotube cell diameters in the four treatment groups (1 ng/mL, 10 ng/mL, 100 ng/mL, and 1000 ng/mL) were significantly larger than model group (*p* < 0.05). This indicates that these concentrations effectively alleviated the atrophy of L6 myotube cell diameters. Among the tested doses, 10 ng/mL was identified as the optimal concentration. These findings suggest that PsP extracted using the UAE-DESs method can mitigate PA-induced oxidative stress in L6 myotube cells, at specific concentrations.

### 3.10. Determination of MHC Protein Expression Level

**Figure 10:**
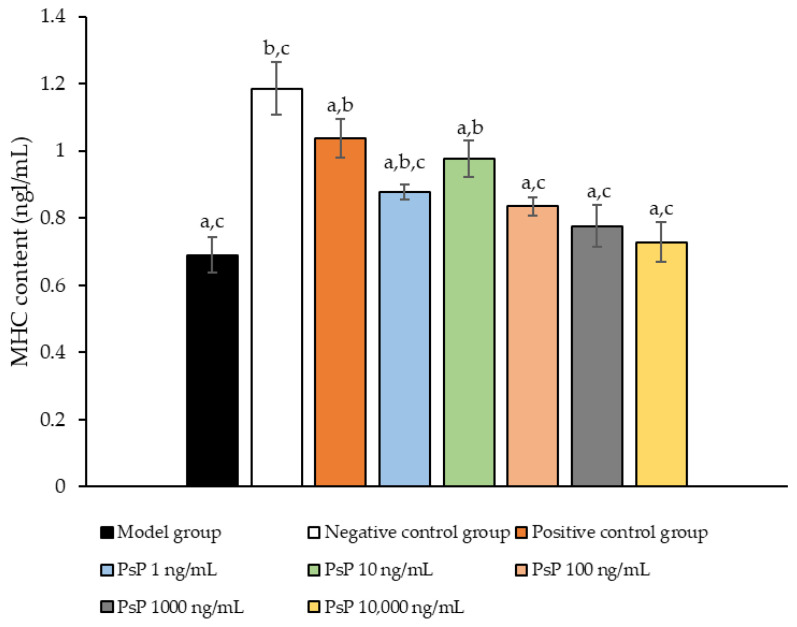
Determination of the expression levels of MHC protein in L6 myotube cells. Note: The superscript “a” indicates a statistically significant difference compared to the negative control group (*p* < 0.05); “b” denotes a statistically significant difference compared to the model group (*p* < 0.05); and “c” represents a statistically significant difference compared to the positive control group (*p* < 0.05).

Figure 10 illustrates the comparison of MHC protein expression levels among the experimental groups. The results revealed that the MHC protein expression levels in the two treatment groups (1 ng/mL and 10 ng/mL) were significantly higher than model group (*p* < 0.05). This suggests that both 1 ng/mL and 10 ng/mL doses effectively enhanced MHC protein expression in L6 myotube cells, with 10 ng/mL being identified as the optimal concentration. These findings indicate that PsP extracted using the UAE-DESs method can improve the capacity of L6 myotube cells to counteract PA-induced oxidative stress, at specific concentrations.

### 3.11. Determination of MyoD Protein Expression Level

**Figure 11:**
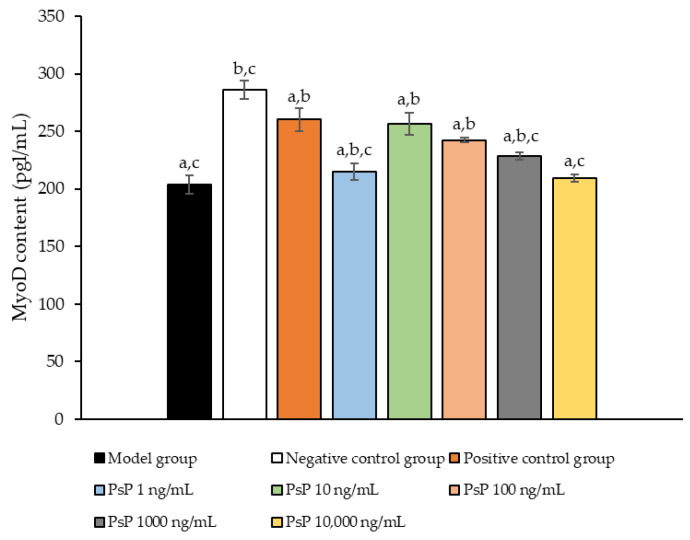
Determination of the MyoD protein expression levels in L6 myotube cells. Note: The superscript “a” indicates a statistically significant difference compared to the negative control group (*p* < 0.05); “b” denotes a statistically significant difference compared to the model group (*p* < 0.05); and “c” represents a statistically significant difference compared to the positive control group (*p* < 0.05).

Figure 11 presents a comparison of MyoD protein expression levels across the experimental groups. The results demonstrated that the MyoD protein expression levels in the four treatment groups (1 ng/mL, 10 ng/mL, 100 ng/mL, and 1000 ng/mL) were significantly higher than model group (*p* < 0.05). This indicates that these concentrations effectively enhanced the MyoD protein expression in L6 myotube cells, with 10 ng/mL being identified as the optimal dose. These findings suggest that PsP extracted using the UAE-DESs method can improve the ability of L6 myotube cells to counteract PA-induced oxidative stress, at specific concentrations.

## 4. Discussion

It has been well documented that PA can induce oxidative stress in cells, and several studies have utilized PA to establish an SO model characterized by ectopic lipid deposition and muscle atrophy [26]. The L6 cell line, derived from rat skeletal muscle, is widely employed in muscle-related research due to its high proliferation capacity, stable growth characteristics, and suitability for long-term cell culture. Furthermore, L6 cells retain many functional and phenotypic features of muscle cells, making them a valuable model for studying muscle-related diseases, injuries, and associated mechanisms. In this study, PA was used to treat L6 cells in order to establish an oxidative stress cell model simulating SO, aiming to provide preliminary technical support for subsequent investigations into the effects and mechanisms of PsP in ameliorating SO. The results confirmed the successful construction of an oxidative stress model in L6 cells induced by PA, validating its utility for further research.

NAC is known for its potent antioxidant properties, which enable it to directly scavenge free radicals, enhance the body’s antioxidant capacity, and reduce the production of inflammatory cytokines, chemokines, and adhesion molecules [31,32]. Additionally, NAC has been shown to mitigate oxidative stress-induced cellular damage and senescence. Given these properties, NAC was selected as the positive control in this study to evaluate the efficacy of PsP extracted using the UAE-DESs method. The results demonstrated that NAC effectively improved the antioxidant capacity of L6 cells against oxidative stress induced by PA, simulating SO. This supports its role as a reliable reference in assessing the antioxidant effects of PsP in this experimental model.

The cell proliferation activity assay conducted in this study demonstrated that PsP enhances the resistance of L6 cells, delaying the decline in cell proliferation activity. The effect can likely be attributed to the ability of polysaccharides to inhibit ectopic lipid deposition, thereby reducing the generation of oxygen free radicals and minimizing oxidative damage to cell membranes and DNA. This mechanism helps preserve cellular structural integrity [33]. Additionally, the anti-inflammatory properties of polysaccharide can reduce the release of inflammatory cytokines, lowering inflammation levels and alleviating inflammatory responses. Furthermore, polysaccharides can modulate intracellular signaling pathways, positively influencing cell proliferation and the expression of apoptosis-related proteins, thereby promoting cell growth [34,35]. These findings and analyses suggest that PsP extracted using the UAE-DESs method can retain the functional properties of conventional polysaccharides. In subsequent mechanistic studies, we will further investigate and validate these potential mechanisms.

Lipid ectopic deposition occurs when the excessive production of free fatty acids surpasses the storage capacity of adipose tissue, leading to the abnormal accumulation of fatty acids in non-adipose tissues such as skeletal muscle. Studies have shown that excessive intracellular lipid accumulation can directly induce oxidative stress [36,37]. In this study, the TG results indicated that the optimal dose of PsP was 10 ng/mL, a level which effectively enhanced the resistance of L6 cells to the oxidative stress induced by PA. This finding was further supported by oil red O staining results, confirming that PsP can mitigate oxidative stress by improving the ability of L6 cells to resist lipid ectopic deposition. Research has demonstrated that ectopic lipid deposition in skeletal muscle and other tissues can contribute to the development of SO [38]. Therefore, reducing the occurrence and progression of ectopic lipid deposition in muscle is crucial for the prevention and treatment of SO [39]. The TG results from this study suggest that PsP extracted using the UAE-DESs method not only enhances the resistance of L6 cells to lipid ectopic deposition, but also holds potential for preventing and treating SO in skeletal muscle.

After confirming that PsP enhances the resistance of L6 cells to ectopic lipid deposition, this study further investigated the impact of PsP on oxidative stress. Oxidative stress occurs when excessive reactive ROS and other harmful substances disrupt the balance between oxidation and antioxidant systems, leading to oxidative damage. Excessive ROS not only directly inhibits the activity of mitochondrial respiratory-chain enzyme complexes but also causes oxidative damage to mitochondrial DNA, proteins, and lipids, further increasing ROS levels and creating a vicious circle that exacerbates oxidative stress. The ROS detection results in this study demonstrated that the optimal dose of PsP was 10 ng/mL, which significantly enhanced the resistance of L6 cells to PA-induced oxidative stress. This finding suggests that PsP extracted using the UAE-DESs method can, at appropriate concentrations, improve the antioxidative capacity of L6 cells. The human enzymatic defense system, primarily composed of GSH-Px, SOD, and CAT, plays a critical role in combating oxidative stress [40]. GSH-Px scavenges hydrogen peroxide and lipid peroxides, SOD specifically eliminates superoxide anion free radicals and enhances immunity against free radical-related diseases, and CAT breaks down intracellular hydrogen peroxide, preventing tissue damage caused by excessive hydrogen peroxide levels [41]. To explore whether PsP could provide a more comprehensive protective effect in L6 cells, we analyzed the activities of these three key antioxidant enzymes. The results revealed that the optimal dose of PsP was 10 ng/mL, which effectively enhanced the resistance of L6 cells to PA-induced oxidative stress. This effect may be attributed to nuclear factor related factor 2 (Nrf2), a crucial transcription factor that regulates the expression of GSH-Px, SOD, and CAT. Previous research has suggested that astragalus polysaccharides can enhance myocardial antioxidant capacity and reduce oxidative stress, potentially through the regulation of the Keap1/Nrf2-ARE signaling pathway [42]. Therefore, PsP extracted using the UAE-DESs method might similarly promote the activities of these three antioxidant enzymes by modulating Nrf2. In subsequent studies, we will further investigate the effect of PsP on this signaling pathway to elucidate its underlying mechanisms.

MDA, a byproduct of cell membrane lipid peroxidation, is widely used as a biomarker to assess oxidative stress levels and the extent of cellular damage [43]. In this study, the optimal dose of PsP for reducing MDA levels was determined to be 10 ng/mL. This indicates that 10 ng/mL of PsP is the most effective concentration for enhancing the resistance of L6 cells to PA-induced oxidative stress and cell injury. These findings suggest that PsP extracted using the UAE-DESs method can enhance the protection of L6 cells against lipid oxidative damage in skeletal muscle. This implies that PsP may play a role in strengthening skeletal muscle and mitigating oxidative stress by improving cellular antioxidant capacity and reducing the generation of reactive oxygen species.

Oxidative stress in skeletal muscle not only promotes protein degradation but also inhibits protein synthesis, thereby accelerating the onset and progression of muscle atrophy. Powers et al. [44] demonstrated that oxidative stress inhibits the AKt/mTORC1 signaling pathway, with the inhibitory effect intensifying as ROS levels increase, ultimately leading to reduced protein synthesis in skeletal muscle. Dodd et al. [45] revealed that excessive ROS can activate both the autophagy–lysosome and ubiquitin–proteasome systems, contributing to skeletal muscle protein degradation. This process may be mediated by ROS regulation of the NF-kB and FOXO transcription factors. Additionally, other studies have shown that skeletal muscle protein degradation can also be linked to the activation of caspase-3 protease and calpain by ROS, a process which results in further muscle atrophy.

Regarding the sources of ROS, Choi and Nicolas et al. [46,47] demonstrated that PA is a significant contributor, as it can elevate oxidative stress levels in C2C12 myotube cells. The accumulation of ROS induced by PA, along with lipid metabolic intermediates in muscle cells, creates a lipotoxic environment that damages skeletal muscle cells and leads to muscle atrophy. This process results in reductions in MHC expression and myotube diameters. Their findings suggest that PA can induce muscle cell atrophy. In our study, the results indicated that PsP extracted using the UAE-DESs method can protect L6 cells from PA-induced damage, highlighting its potential to mitigate the adverse effects of PA on skeletal muscle cells.

MHC is a fundamental component of myosin, playing a critical role in maintaining the normal function of muscle cells. MyoD, a key regulatory factor, promotes muscle cell differentiation [48] and is essential for muscle development, the differentiation of stem cells into muscle cells, and muscle regeneration. In this study, we evaluated the diameter of myotube cells and the expression levels of MHC and MyoD proteins to investigate whether PsP could mitigate L6 cell atrophy by enhancing the L6 cells’ resistance to PA-induced oxidative stress. The results demonstrated that PsP extracted by UAE-DESs significantly enhanced the resistance of L6 cells, reducing the atrophy of myotube cell diameter. This protective effect can be attributed to the free scavenging ability of PsP, which mitigates oxidative stress, promotes muscle cell metabolism, enhances cell vitality and function, reduces cellular damage, and ultimately inhibits the atrophy of cell diameters.

Furthermore, our results demonstrated that the optimal dose of PsP (10 ng/mL) effectively enhanced the resistance of L6 cells in cases of reductions in MHC and MyoD protein expression. During muscle injury and repair processes, the expression levels of MHC and MyoD can undergo significant changes. For instance, MHC expression often increases following muscle injury to promote muscle fiber repair and regeneration, while MyoD expression typically rises markedly in the early stages of injury recovery, facilitating muscle cell regeneration and differentiation. Therefore, depending on the type of intervention, its purpose, and the mechanisms influencing muscle formation and development, distinct differences in MHC and MyoD expression may be observed under various intervention conditions. These findings align with previous research which showed that paeoniflorin can inhibit PA-induced myotube cell protein degradation and muscle cell atrophy by activating the AKT/mTOR pathway [49]. The optimal dosage for each indicator was consistently observed to be 10 ng/mL, likely because lower concentrations of PsP provide an environment favorable for maintaining stable and active cell conditions. However, due to the acidic polysaccharide properties of L6 cells, excessively high concentrations of PsP may alter the pH of their environments, leading to intracellular changes and subsequent declines in related indicators. Alternatively, it is possible that PsP, at higher concentrations, exhibits certain toxic effects on L6 cells. Based on the above analysis and discussion, it can be concluded that PsP extracted using the UAE-DESs method enhances the resistance of L6 cells to PA-induced atrophy while increasing the extraction efficiency of PsP. This indicates that PsP extracted by UAE-DESs retains the antioxidant properties of polysaccharides. The likely reason is that the UAE-DESs method does not compromise the structural integrity or physiological activity of PsP, that is further supported by our previous studies. These studies demonstrated that the molecular weights, monosaccharide composition, and infrared spectra of PsP extracted using the UAE-DESs method were comparable to those obtained through conventional ultrasound extraction. Therefore, it can be inferred that the UAE-DESs method preserves the structure and bio-activity levels of polysaccharides [30,50,51].

## 5. Conclusions

This study demonstrated that PsP extracted using the UAE-DESs method retains the antioxidant properties of polysaccharides, even as the extraction efficiency is enhanced. This indicates that the UAE-DESs method does not compromise the functional integrity of PsP. Furthermore, this study confirmed that PsP extracted by the UAE-DESs method can improve the antioxidant ability of L6 cells against PA-induced oxidative stress, providing a valuable research foundation and a highlighting the potential of PsP in the prevention and treatment of SO.

## Figures and Tables

**Table 1:** Reagents used in this research.

Name of Product	Product Code	Company (City, State, Country)
DEME culture medium	SH30243.01	Thermo Fisher Scientific, Inc. (Waltham, MA, USA)
Fetal bovine serum	10091148	Thermo Fisher Scientific, Inc. (Waltham, MA, USA)
Horse serum	16050122	Thermo Fisher Scientific, Inc. (Waltham, MA, USA)
Dual antibodies (penicillin + streptomycin)	15140122	Thermo Fisher Scientific, Inc. (Waltham, MA, USA)
Palmitic acid	P0500	Sigma Co., Ltd. (Louis, MO, USA)
N-Acetyl-L-cysteine	A7250	Sigma Co., Ltd. (Louis, MO, USA)
CCK-8 cell activity detection kit	C0037	Beyotime, Inc. (Shanghai, China)
Reactive oxygen species detection kit	S0033S	Beyotime, Inc. (Shanghai, China)
Glutathione peroxidase activity detection kit	BC1195	Beijing Solarbio Life Science, Inc. (Beijing, China)
Total superoxide dismutase activity detection kit	S0101S	Beyotime, Inc. (Shanghai, China)
Catalase activity detection kit	BC0205	Beijing Solarbio Life Science, Inc. (Beijing, China)
Lipid oxidation detection kit	S0131M	Beyotime, Inc. (Shanghai, China)
Triglyceride determination kit	A110-1-1	Nanjing Jiacheng Bioengineering Institute, Inc. (Nanjing, China)
Oil red O dyeing solution	D027-1-3	Nanjing Jiacheng Bioengineering Institute, Inc. (Nanjing, China)
Rat myosin heavy chain enzyme linked immunosorbent assay kit	LV20904	Animaluni, Inc. (Shanghai, China)
Rat myogenic differentiation protein enzyme-linked immunosorbent assay kit	ELK6019-96	ELK Biotechnology Co., Ltd. (Denver, CO, USA)

**Table 2:** Treatment and modeling regimen for L6 cells.

Group	Intervention Scheme	Modeling Scheme
Model group	Differentiation medium	Differentiation medium containing 0.75 mMPA
Negative control group (Blank control group)	Differentiation medium	Differentiation medium
Positive control group	Differentiation medium containing 5 mM NAC	Differentiation medium containing 0.75 mM PA
Dose group 1	Differentiation medium containing 1 ng/mL of PsP extracted with the UAE-DESs method	Differentiation medium containing 0.75 mM PA
Dose group 2	Differentiation medium containing 10 ng/mL of PsP extracted with the UAE-DESs method	Differentiation medium containing 0.75 mM PA
Dose group 3	Differentiation medium containing 100 ng/mL of PsP extracted with the UAE-DESs method	Differentiation medium containing 0.75 mM PA
Dose group 4	Differentiation medium containing 1000 ng/mL of PsP extracted with the UAE-DESs method	Differentiation medium containing 0.75 mM PA
Dose group 5	Differentiation medium containing 10,000 ng/mL of PsP extracted with the UAE-DESs method	Differentiation medium containing 0.75 mM PA

## Data Availability

The original contributions presented in this study are included in the article. Further inquiries can be directed to the corresponding authors.
